# Research Priorities in Perioperative Nursing Across the Lifespan: An e-Delphi Consensus Study

**DOI:** 10.3390/nursrep16080273

**Published:** 2026-08-04

**Authors:** Ana Ramos, Rita Maurício, Sara Pires, Susana Mendonça, Lúcia Jerónimo, Hélder Fonseca, Piedade Pinto, Anabela Carvalho, Idalina Gomes, Eunice Sá, Carla Nascimento

**Affiliations:** 1 Comprehensive Health Research Centre (CHRC), University of Évor, 7000-811 Évora, Portugal; susana.mendonca@enfermagem.ulisboa.pt; 2 Nursing Research Innovation and Development Centre of Lisbon (CIDNUR), School of Nursing, Universidade de Lisboa, 1649-004 Lisbon, Portugal; mauricio@enfermagem.ulisboa.pt (R.M.); sarapires@enfermagem.ulisboa.pt (S.P.); luciajeronimo@enfermagem.ulisboa.pt (L.J.); idgomes@enfermagem.ulisboa.pt (I.G.); esa@enfermagem.ulisboa.pt (E.S.); carla.nascimento@enfermagem.ulisboa.pt (C.N.); 3 Local Health Unit of Lezírias, 2005-177 Santarém, Portugal; 4 Local Health Unit of São José, 1150-199 Lisbon, Portugal; helder.lopes@ulssjose.min-saude.pt; 5 Local Health Unit of Médio Tejo, 2350-754 Torres Novas, Portugal; piedade.pinto@ulsmt.min-saude.pt (P.P.); anabela.valente@ulsmt.min-saude.pt (A.C.)

**Keywords:** perioperative nursing, e-Delphi, nursing research, lifespan

## Abstract

**Background/Objective:** Perioperative nursing care encompasses a diverse patient population with varying needs, anatomical and physiological characteristics, and differing levels of vulnerability, demanding specialized knowledge and skills. This study aims to identify key research priorities in perioperative nursing across the lifespan. **Methods:** A two-round e-Delphi study was conducted across three Portuguese hospitals, involving expert perioperative nurses (87 in the first round and 69 in the second). Participants rated the importance of various research topics on a 5-point Likert scale about six dimensions: (1) professional development and non-technical skills, (2) person-centered care, (3) organizational aspects and work environment, (4) patient and team safety, (5) digital innovation, and (6) patient outcomes and quality of care. Data were analyzed using mean scores, Content Validity Ratio (CVR) for initial validation, and the Coefficient of Variation (CV) to determine the level of consensus. The reporting of this study followed the DELPHISTAR (Guideline for Reporting Delphi Studies) recommendations. **Results:** A high degree of consensus was achieved (all CV < 0.20). The experts established a consensus on 48 components. The highest-rated priority was nurse burnout and professional satisfaction (Mean = 4.74). This was closely followed by patient safety (Mean = 4.68), team safety (Mean = 4.65), disaster response (Mean = 4.62), and emergency management (Mean = 4.58). Acute pain control and interdisciplinary communication also emerged as critical areas. Topics such as digital innovation and artificial intelligence, while valued, received lower priority scores. **Conclusions:** The identified priorities should direct funding toward research, education, quality improvement, and health policies. Future studies should focus on developing interventions to promote a favorable perioperative environment and optimize clinical responses to individuals’ conditions, catastrophes, and life-threatening situations across the lifespan.

## 1. Introduction

Global demographic transformations, particularly accelerated population ageing, constitute one of the major structural challenges facing health systems in the twenty-first century [1,2]. This phenomenon is especially pronounced in European countries, where population ageing has assumed structural and largely irreversible characteristics. In Portugal, nearly one quarter of the population is already aged 65 years or older, placing the country among the most aged in the European Union and intensifying pressure on the sustainability of health systems, service organization, and health workforce management [3,4].

Demographic ageing has occurred in parallel with an increasing prevalence of chronic diseases and multimorbidity, shaping a new epidemiological paradigm dominated by long-term conditions of high clinical complexity. Cardiovascular diseases, diabetes mellitus, chronic respiratory diseases, oncological, musculoskeletal, and neurodegenerative conditions currently represent the leading causes of morbidity, mortality, and functional disability worldwide [1,4]. The coexistence of multiple conditions is frequently associated with polypharmacy, an increased risk of adverse events, clinical frailty, and progressive loss of functional autonomy, particularly among older and socially vulnerable populations [5].

A substantial component of the therapeutic response to these conditions involves surgical care, integrated into care pathways with curative, palliative, preventive, or symptom-control objectives. Surgery constitutes a cornerstone of modern health systems and is essential in the management of multiple chronic conditions, cancer control, trauma care, acute illness, and interventions aimed at improving quality of life [6,7].

Even in palliative contexts, surgical interventions and perioperative care may play a meaningful role in pain relief, reduction in suffering, and enhancement of overall well-being, requiring highly individualized and ethically grounded approaches [8]. Perioperative nursing ensures safe, continuous, integrated, and person-centred care for individuals undergoing surgery. Perioperative nursing encompasses a set of specialized interventions delivered across the preoperative, intraoperative, and postoperative phases, aimed at risk assessment, complication prevention, patient safety, and optimization of health outcomes [9,10].

Perioperative care is associated with significant clinical risks not only in older adults but in children, pregnant women, and other vulnerable phases of the life course. During the surgical experience, the occurrence of anesthetic complications, hemorrhage, healthcare-associated infections, and prolonged recovery requires specialized technical, relational, and ethical competencies from nursing teams [11]. Concurrently, perioperative nursing care has been influenced by rapid technological advancements and the digital transformation of health systems. The integration of electronic health records, advanced monitoring systems, clinical decision support tools, telemonitoring, and artificial intelligence–based applications offers substantial potential for risk stratification, early detection of complications, personalization of care, and improved efficiency of care processes [12,13,14]. However, these advances also raise significant challenges related to system interoperability, cybersecurity, professional training, integration into real-world clinical workflows, and the evaluation of their actual impact on health outcomes, reinforcing the need for rigorous and translational research [15].

Despite the clinical, organizational, and social relevance of perioperative nursing, substantial gaps in consensus on research priorities that can inform health frameworks, resource planning, and specialized education programmes [16,17]. International professional organizations and consensus-based studies have identified priority areas including patient safety, pain management, infection prevention, humanization of care, professional well-being, clinical ethics, and the integration of emerging technologies, highlighting shared needs across diverse geographical contexts and health system settings [18,19]. Accordingly, this e-Delphi study aims to establish a consensus on perioperative nursing research priorities across the lifespan within the Portuguese context, thereby informing the development of a strategic agenda for clinical practice, educational curricula, and health policies.

## 2. Methods

### 2.1. Design

A methodological, consensus-building study was conducted using the e-Delphi technique [20]. The e-Delphi is a digital adaptation of the classic Delphi method, which aims to systematically reach a reliable consensus among a panel of experts through successive rounds of questionnaires and controlled feedback [21,22]. By transitioning from traditional postal mailouts to online platforms, the e-Delphi maintains the core principles of anonymity, iteration, and statistical response while enhancing data-collection efficiency and removing geographical barriers [23]. The entire design, execution, and tracking of the iterative rounds strictly adhered to the DELPHISTAR (Guideline for Reporting Delphi Studies) statement to guarantee comprehensive reporting transparency [24] (Supplementary File S1). Although the study protocol was not publicly pre-registered, the complete study design and data collection instruments were established a priori. The study was approved by the Ethics Committees of the participating hospital units before its commencement.

### 2.2. Participants

A purposive sampling strategy was employed to recruit a panel of expert perioperative nurses. To ensure institutional and geographical diversity, a total of 250 perioperative nurses were initially invited to participate. These professionals were recruited from three distinct hospital units within the Lisbon and Tagus Valley health region: one major university hospital located in the Lisbon metropolitan area, and two regional district hospitals in Portugal.

To ensure high-quality and informed clinical consensus, inclusion criteria required panelists to possess: (a) a minimum of five years of active professional experience in nursing practice [25], and (b) current clinical placement in one or more of the following specialized perioperative environments: Operating Room (OR), Post-Anesthesia Care Units (PACU), Post-Surgery Inpatient Units, Surgical Intensive Care Units (SICU), or Ambulatory Surgery Centers. Eligible nursing experts were formally invited via e-mail, which detailed the study’s objectives, ethical assurances of anonymity, and the voluntary nature of participation.

The literature indicates that a panel comprising approximately 8 to 23 members is generally sufficient to yield meaningful and actionable results [21]; a substantially larger panel was deliberately targeted to capture the wide clinical, geographic, and organizational diversity of perioperative nursing settings. Out of the 250 perioperative nurses initially invited, a total of 87 nursing experts voluntarily agreed to participate and completed the first round of the Delphi process (*n* = 87), yielding an initial response rate of 34.8%. In the subsequent round, 69 nurses remained actively engaged and completed the entire evaluation (*n* = 69). This demonstrated a robust retention rate of 79.31% between rounds, with an attrition rate well within the acceptable methodological thresholds for multi-round consensus studies. The comprehensive socio-demographic and professional characteristics of the panel across both rounds are detailed in Table 1.

Regarding age, the 41–45 cohort predominated in Round 1 (25.58%), shifting to the ≥51 cohort in Round 2 (36.23%). The panel was predominantly female (Round 1: 73.6%; Round 2: 69.6%). Academically, Master’s degrees comprised 26.43% in Round 1 and 26.09% in Round 2, while specialized postgraduate certifications accounted for 17.24% and 23.19%, respectively. Professionally, Specialist Nurses represented the vast majority (Round 1: 78.16%; Round 2: 81.16%), whereas General Care Nurses constituted 21.84% and 18.84%, respectively. The panel’s clinical experience was extensive, reflected by a mean of 21.96 years (SD = 8.21) in Round 1 and 23.34 years (SD = 7.50) in Round 2.

### 2.3. Data Collection and Procedure

A two-round electronic Delphi (e-Delphi) survey was executed to establish clinical consensus regarding perioperative nursing research priorities. Before baseline data collection, formal electronic informed consent was obtained from all participating expert nurses who agreed to join the panel. The multi-round Delphi architecture was systematically rolled out through secure, individualized e-mail correspondence, preventing mutual peer contamination, and facilitated an agile feedback infrastructure. The study was conducted sequentially between April and June 2025.

The structural matrix of the initial e-Delphi questionnaire was mapped from a comprehensive synthesis of the framework established by the Association of PeriOperative Registered Nurses (AORN) Research Priorities 2023–2028 [26]. Based on this framework, the emergent instrument was organized into six distinct dimensions encompassing perioperative nursing research priorities across the entire lifespan (from pediatrics to older adulthood): (1) professional development and non-technical skills; (2) person-centered care; (3) organizational aspects and the work environment; (4) patient and team safety; (5) digital innovation; and (6) patient outcomes and quality of care.

**Figure 1:**
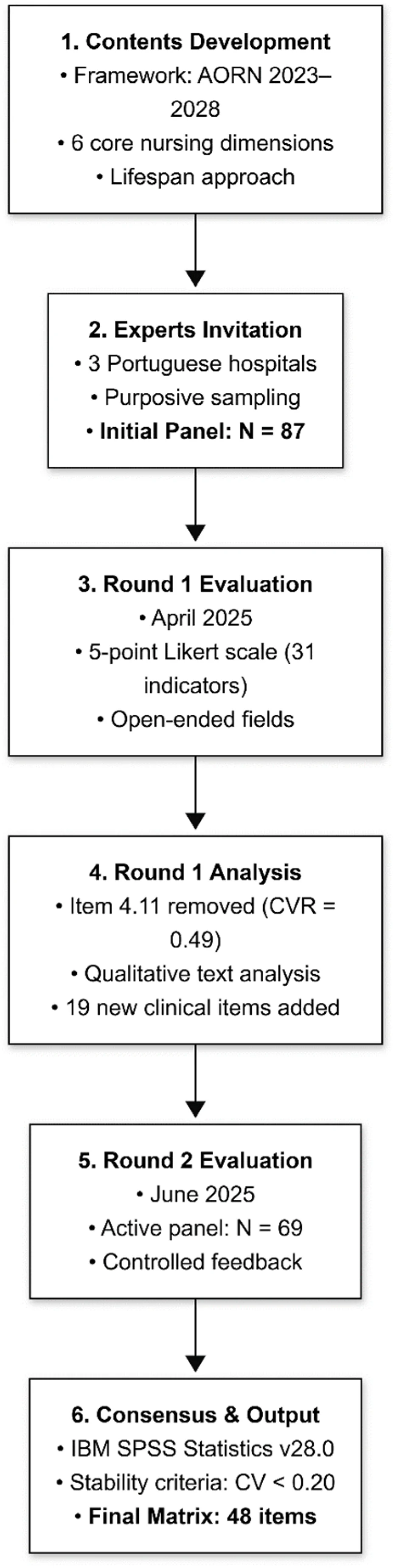
Flowchart of the Delphi method step.

A two-round e-Delphi design was established a priori to achieve consensus while minimizing panel attrition and response fatigue (Figure 1). A third round was planned as a contingency if consensus criteria (CV < 0.20) were not met after Round 2. In Round 1, panelists appraised the priority level of each research topic using a 5-point Likert scale. In Round 1, open-ended fields allowed panelists to provide qualitative feedback, structural revisions, and additional priority items. Qualitative responses were analyzed using content analysis. Two investigators independently coded the data, and discrepancies were resolved through consensus with a third senior researcher. Newly identified items and modifications were synthesized into standardized statements, categorized under the six dimensions, and incorporated into the Round 2 questionnaire alongside Round 1 statistical feedback (mean, SD, and CVR). In Round 2, experts were provided with a controlled feedback report. This report integrated the panel’s aggregate quantitative metrics from the previous iteration alongside a synthesized summary of the qualitative feedback. Experts then systematically re-evaluated and adjusted their ratings against the group’s distribution, driving a structured convergence toward final consensus.

### 2.4. Data Analysis

Quantitative data were collected using a 5-point Likert scale ranging from 1 (not at all important to investigate) to 5 (very important to investigate). To measure central tendency, dispersion, and establish expert consensus from the quantitative data, descriptive and inferential statistics were computed. For each research priority component, the mean score, standard deviation (SD), Content Validity Ratio (CVR), and Coefficient of Variation (CV) were calculated across both rounds.

The CVR was utilized for the initial validation of the components, representing a linear transformation of the proportion of experts who scored an item within the positive priority threshold (scores of 4 or 5, signifying “important” or “very important” to investigate) [27]. The CVR was calculated using the following formula:

$$CRV=\frac{n_{e}-\frac{N}{2}}{\frac{N}{2}}$$

where n_e_ represents the number of expert nurses responding with a score of 4 or 5, and N represents the total number of experts in that specific round (N = 87 in Round 1; N = 69 in Round 2). The CVR values range from −1.0 to +1.0. A positive CVR indicates that more than half of the panel evaluated the topic as a priority for research; a value of zero implies exactly half of the panel agreed, and a negative value means less than half categorized it as a priority.

To evaluate the stability and consensus level of the responses across the iterations, the CV was calculated as follows [28]:

$$CV=\frac{SD}{Mean}$$

A CV threshold (CV < 0.20) was defined as the criterion for agreement. Due to the process where items were refined, dropped, or added between rounds, consensus was evaluated using metrics rather than paired comparisons. This cutoff aligns with the agreement threshold of 80% among panelists to establish consensus. The CV was selected as the stopping criterion to evaluate consensus and consistency. As a metric of dispersion, the CV normalizes variance against the mean, providing a measure of convergence across rounds [29]. Qualitative data gathered from the open-ended fields in Round 1 underwent content analysis to refine existing statements, remove redundant elements, or add newly suggested clinical topics. In Round 2, controlled feedback was operationalized by providing the panel with an aggregate summary of the previous round’s quantitative metrics and qualitative suggestions, allowing individual experts to anonymously re-evaluate and calibrate their priority ratings against the group’s distribution. All statistical analyses were executed using IBM SPSS Statistics for Windows, version 28.0 (IBM Corp., Armonk, NY, USA).

## 3. Results

### 3.1. Round 1

The first round of the e-Delphi survey was dedicated to assessing the initial set of items derived from the literature and structural frameworks. While the vast majority of the proposed indicators demonstrated a high degree of consensus and strong content validity, granular adjustments were required based on both statistical thresholds and qualitative expert feedback. Specifically, Item 4.11., “Use of surgical caps and shoe covers in specific perioperative practices” failed to meet the minimum acceptable consensus criteria established by the panel (CVR = 0.49), leading to its permanent exclusion from the matrix at this stage.

At the same time, qualitative analysis of the open-ended fields allowed experts to suggest structural refinements. Instead of simplifying complex clinical realities, the panel emphasized expanding the framework to capture emerging challenges in contemporary perioperative nursing. Consequently, several new clinical and organizational items were developed, refined, and integrated into Round 2 (Table 2).

### 3.2. Round 2

In the second Delphi round, the introduction of new items across all domains successfully captured emerging priorities in perioperative nursing, achieving strong validation with Content Validity Ratio (CVR) values ranging from 0.77 to 1.00 and Coefficient of Variation (CV) values well below the 0.5 threshold, indicating high stability and expert consensus. Within Professional Development and Non-Technical Skills, the newly integrated items reflected a clear consensus on advanced practice and crisis management. Interdisciplinary briefing and debriefing in perioperative emergencies (Item 1.3) emerged as a high priority, alongside the definition of core competencies for the expert perioperative nurse.

Regarding Person-Centered Care, the most significant addition was the integration of specific care workflows for individuals with palliative needs (Item 2.6), which reached a high consensus, successfully filling a structural gap in standard perioperative design. The domain of Organizational Aspects and Work Environment saw the inclusion of several metrics. The new indicator for nurse burnout and professional satisfaction (Item 3.10) achieved a high score, indicating that experts view systemic team well-being as a mandatory prerequisite to care quality. Other newly introduced organizational priorities also gathered strong support, including micromanagement (Item 3.3), environmental sustainability (Item 3.8), standard protocols (Item 3.9), nursing-sensitive indicators (Item 3.11), and continuous quality improvement projects (Item 3.12).

In the Patient and Team Safety domain, a subset of new targeted clinical and operational items achieved strong consensus layout: contingency planning/disaster response (Item 4.13), emergency hemodynamic instability (Item 4.8), and healthcare-associated infections (Item 4.15). Standard safety processes: Preoperative assessment visits (Item 4.14), the surgical safety checklist (Item 4.7), perioperative thromboembolism prevention (Item 4.12), and fall prevention (Item 4.6). In terms of Digital Innovation, the newly introduced focus is on artificial intelligence (AI) and innovation (Item 5.3). However, its lower comparative score reveals a panel that is forward-looking yet deliberately cautious regarding immediate technological disruption. Finally, under Patient Outcomes and Quality of Care, two newly integrated items, acute pain management (Item 6.6), and the evaluation of the operating room’s sound environment on the patient’s subjective experience (Item 6.7), were successfully validated, highlighting the panel’s recognition of environmental stressors on patient outcomes.

## 4. Discussion

To analyze the expert consensus and establish a framework for perioperative nursing investigation, the items were stratified into three priority tiers. Ranking was operationalized by ordering all items meeting the content validity ratio inclusion criteria in descending order of their mean scores. The analysis is structured around these three tiers of investigative priority: high, intermediate, and moderate research priority.

### 4.1. High Research Priority (Mean ≥ 4.54)

The expert panel established a distinct consensus regarding high-priority research areas, led by nurse burnout and professional satisfaction. This consensus identifies the psychological integrity and occupational health of the perioperative workforce as a primary area for future empirical investigation. This determination aligns with evidence from Lin et al. [30], which demonstrates that clinician burnout directly correlates with a decline in objective patient safety metrics, overall care quality, and postoperative outcomes. The inclusion of workplace violence/bullying in the perioperative environment as a priority is supported by a European survey [31], which demonstrates that workplace violence is common across all perioperative disciplines, and violence undermines institutional stability, establishing a need for multi-level interventions to prevent harm to both clinical staff and patients.

A convergence was observed in a cluster of safety-related research priorities, specifically patient safety in the perioperative environment, perioperative team safety, and intraoperative materials safety. By evaluating patient, staff, and material safety as closely aligned priorities, the panel indicates that perioperative research must conceptualize safety as an emergent property of the care environment. This perspective interfaces directly with clinical quality and safety, resulting from complex interactions between clinicians, tasks, tools, environments, and organizational architectures [32]. Furthermore, this integrated research direction addresses the operational realities identified by Elbahi et al. [33], who noted that the majority of surgical adverse events originate from cumulative, minor process deviations distributed across the perioperative continuum rather than isolated failures.

The expert panel prioritized non-technical preparation and structured communication as critical domains for future perioperative nursing research. Specifically, the validation of interdisciplinary briefing and debriefing in perioperative emergencies establishes preoperative and postoperative communication protocols as primary areas for clinical investigation and optimization. This selection aligns with empirical evidence from Schaap et al. [34], which indicates that structured briefing and debriefing frameworks enhance the perioperative team climate. Researching these mechanisms is essential to understanding how teams develop acute situational awareness, identify latent clinical risks early, streamline interprofessional collaboration, and maintain operational resilience during surgical crises. This communicative foundation aligns with consensus on human factors and education in perioperative nursing and infection control. The panel’s prioritization of hemodynamic emergency research addresses literature gaps identified by the Anesthesia Patient Safety Foundation [35], specifically regarding data on risk identification metrics and monitoring efficacy.

Vázquez-Calatayud et al. [36] demonstrated that structured education improves technical skill execution and modifies professional attitudes. Perioperative nursing research must focus on mechanisms that empower clinicians to dismantle rigid institutional barriers, thereby linking clinical competence directly to the delivery of individualized care [37]. Multivariate analyses confirm that structured nursing approaches function as independent protective factors against healthcare-associated complications. This highlights the critical need for future nursing research to focus on standardized perioperative care delivery models, ensuring that clinical work environments effectively reduce infectious risks and enhance short-term recovery trajectories [38].

The valorization of perioperative emergencies/hemodynamic instability and contingency planning/disaster response addresses clinical and operational vulnerabilities identified in contemporary safety literature. An umbrella review of 24 studies highlighted that those deficiencies in education, organizational support, technological advancements, psychological readiness, and interprofessional collaboration constitute major systemic barriers that hinder emergency capacity and health system resilience [39]. Correspondingly, the literature on perioperative interventions identifies a primary failure in current practice: the reliance on generalized vigilance rather than structured frameworks. The evidence establishes that early physiological deterioration drives preventable harm due to the absence of system-level architectures. To resolve these operational shortcomings, research must focus on structures that integrate preoperative risk assessment, intraoperative safety processes, and postoperative surveillance with defined escalation pathways, driven by teamwork and a safety culture [33].

### 4.2. Intermediate Research Priority (4.35 ≤ Mean ≤ 4.53)

The intermediate tier focuses on the recovery of functional parameters, the humanization of the surgical environment, sustainability, and the clinical adaptation of care pathways for vulnerable demographics. Expert-valued research priorities in this domain include the adaptation of perioperative care for pediatric, older adults, individuals with cognitive impairment, and patients with palliative needs. The perioperative nursing gap primarily focuses on adult guidelines, which fail to account for the unique physiological and psychological variables of distinct demographic groups. This limitation becomes evident when analyzing age-sensitive perioperative anxiety and stress responses. In pediatric cohorts, preoperative anxiety is primarily driven by separation from caregivers, fear of the unknown, and a lack of cognitive coping mechanisms [40]. Conversely, in the presence of cognitive impairment or geriatric populations, perioperative distress is conditioned by fears regarding the permanent loss of functional independence, unfamiliar environments, and neurocognitive fluctuations [41]. Similarly, for patients with palliative needs, they need a surgical treatment goal targeted at symptom control, dignity maintenance, comfort care, spiritual well-being, and quality of life [42].

In high-pressure, highly technical environments, patient advocacy, intrinsically linked to the promotion of surgical conscience, functions as an active clinical safeguard against the vulnerability of the anesthetized or dependent patient. Advocacy in perioperative care settings plays a critical intermediary role, ensuring that patient safety and fundamental rights are actively preserved despite complex standardized institutional workflows [43,44].

The focus on medication safety research reflects the understanding that intraoperative errors stem primarily from flawed physical workflows, such as poor labeling, syringe presentation defects, and environmental interruptions, rather than isolated individual performance [45] The prioritization of acute pain management interfaces directly with data from a scoping review that identifies gaps in the postoperative period in education, provision of continuity of care, individualized management, support for specific populations, and research and knowledge translation [46]. Aligned with the growing emphasis on perioperative interventions to enhance long-term outcomes [47], the panel established the optimization of symptom control as a core research priority.

In parallel with clinical recovery, the assurance of care quality is anchored in service management, justifying research to improve perioperative care organization models, nurse-to-patient ratios, the availability of standard perioperative protocols, and continuous quality improvement projects within this priority tier, as recommended by Wanyonyi [48].

This risk management perspective and prevention of adverse effects align with the panel’s focus on non-technical skills (communication, teamwork, resilience, and emotional intelligence) and interdisciplinary collaboration. Failures in these areas degrade the safety climate and reflect structural workplace issues [49]. Beyond interpersonal factors, the physical environment also poses risks, encompassing both ecological dimensions, through operating room (OR) environmental sustainability, and acoustic management. OR noise systematically exceeds safety limits, disrupting communication, impairing concentration, and increasing psychosocial stress [50]. Furthermore, due to prolonged shifts, continuous exposure causes staff to habituate to this noise, rendering the hazard structurally invisible [51]. Classifying these relational, ecological, and environmental factors as intermediate research priorities highlights the need to study how acoustic stress, green perioperative practices, team dynamics, and organizational efficiency collectively impact professional performance and the patient’s surgical experience.

### 4.3. Moderate Research Priority (4.07 ≤ Mean ≤ 4.34)

The final tier of the priority matrix focuses on structural frameworks, safety interventions, and technological adjustments. These entries gathered more moderate scores, showing that the panel prioritized immediate clinical safety over exploratory digital tools and administrative designs. This pragmatic stance is clear within the digital innovation cluster, which includes integrated nursing information systems, technology to support clinical decision-making, and the use of artificial intelligence (AI) and innovation in perioperative nursing. Although appropriate, these tools received the lowest priority scores. This cautious approach matches the work of Raposo et al. [52], which highlights that digital literacy and ethical awareness are mandatory prerequisites for AI adoption. Gong et al. [13] reinforce this, noting that most AI applications in perioperative nursing still lack large-scale clinical validation.

A similar focus appears in the moderate prioritization of managerial and procedural metrics. Structural items, core competencies for the expert perioperative nurse, and perioperative nursing-sensitive indicators were grouped with operational concerns, specifically micromanagement in the operating room. The panel clustered these organizational factors alongside safety practices, such as the Surgical Safety Checklist, the preoperative assessment/visit, and essential preventive protocols: perioperative fall prevention, pressure ulcer prevention, and perioperative thromboembolism prevention [53,54,55,56]. Furthermore, patient positioning represents a critical safety intersection between intraoperative care and pressure injury prevention. Meta-analyses show that extreme surgical positioning (e.g., the Trendelenburg position) causes a substantial proportion of pressure and peripheral nerve injuries, with incidence rates rising in specialized surgeries [57,58].

Finally, this tier outlines targets for clinical recovery, equity, and humanization. It incorporates primary rehabilitation goals, the recovery of functional capacity, and the recovery of self-care, alongside physiological stability through the maintenance of perioperative normothermia [59,60]. Furthermore, it addresses equity through the accessibility of perioperative care and clinical adaptations for specific populations, including the adaptation of perioperative care for pregnant women and diverse cultures. The preservation of uteroplacental perfusion requires the avoidance of maternal hypoxaemia, hypotension, hypercapnia, hypocapnia, temperature extremes, and stress, which demands specialized care pathways [61]. Concurrently, the adaptation of care to diverse cultures also received lower priority scores, indicating areas where research needs to explore how to integrate cultural or spiritual dimensions of perioperative care [62].

### 4.4. Limitations

The sample was recruited exclusively from three Portuguese hospitals. Although these institutions provide highly specialized perioperative care, the results reflect the structural, cultural, and organizational reality of a specific national context, which may restrict the direct generalization of these research priorities to national healthcare systems. The initial response rate of 34.8% (87/250) introduces the potential for self-selection bias, as nurses who elected to participate may have possessed greater interest, engagement, or clinical specialization in perioperative research than non-responders. In this study, participation decreased from 87 expert perioperative nurses in the first round to 69 in the second. While the remaining sample size is strong and methodologically sound for achieving stable consensus, this dropout rate could introduce subtle selection biases.

Additionally, certain items (such as Item 3.10) were newly identified through expert suggestions in Round 1 and evaluated for consensus solely in Round 2. Although these items met the a priori consensus threshold, they did not undergo the iterative re-evaluation process with group feedback typical of a full multi-round Delphi process. This methodological distinction should be considered when interpreting these findings. Furthermore, the expert panel consisted entirely of perioperative nurses. While this ensures a deep, specialized understanding of the six investigated dimensions, it omits the perspectives of other vital stakeholders, such as surgeons, anesthesiologists, and patients, who might prioritize different research targets across the lifespan.

## 5. Conclusions

This study establishes a consensus-driven roadmap for perioperative nursing research across the lifespan, demonstrating a hierarchy of clinical and operational priorities. At the top of expert concern, the scores attributed to nurse burnout and professional satisfaction, followed by patient and team safety, indicate that workforce well-being and safety environments are prerequisites for clinical safeguards. This focus aligns with the demand for competencies in managing perioperative emergencies, hemodynamic instability, and contingency planning or disaster response, which outranked long-term avenues like artificial intelligence. This preference highlights the panel’s priority for clinical resilience and structured protocols during life-threatening events. Rather than signaling a stance against technological transition, it emphasizes the immediate necessity of crisis preparedness in frontline healthcare settings.

Consequently, these findings provide a basis for the update of academic and professional nursing curricula. Educational frameworks need to expand beyond standardized guidelines to integrate training in disaster nursing, emergency briefing and debriefing protocols, workforce resilience strategies, and demographic-specific care across the lifespan. Ultimately, while digital innovation remains a path for development, scientific funding, quality improvement initiatives, and educational curricula need to target occupational health, acute symptom control, and institutional disaster readiness to guarantee patient safety and human dignity under pressure.

## Figures and Tables

**Table 1:** Sociodemographic and professional characteristics of the expert panel across e-Delphi rounds.

Variables	Round 1 (*N* = 87)	Round 2 (*N* = 69)
Age (years)		
26–30	5 (5.81%)	1 (1.45%)
31–35	9 (10.47%)	2 (2.90%)
36–40	18 (20.93%)	7 (10.14%)
41–45	22 (25.58%)	11 (15.94%)
46–50	11 (12.79%)	11 (15.94%)
51 ou mais anos	21 (24.42%)	25 (36.23%)
Gender		
Female	64 (73.6%)	48 (69.6%)
Male	23 (26.4%)	21 (30.4%)
Academic Qualifications		
Bachelor’s Degree	49 (56.3%)	35 (50.7%)
Master’s Degree	23 (26.4%)	18 (26.1%)
Postgraduate/Specialization	15 (17.2%)	16 (23.2%)
Professional Category		
Specialist Nurse	68 (78.2%)	56 (81.2%)
Registred Nurse	19 (21.8%)	13 (18.8%)
Professional Experience		
Years of Practice (Mean)	21.96	23.34
Standard Deviation (SD)	8.21	7.5

**Table 2:** Content validation and expert consensus on priorities and components of perioperative nursing research (Round 1 and Round 2).

Priorities and Components *	Round 1			Round 2		
	Mean ± SD	CVR	CV	Mean ± SD	CVR	CV
1. Professional Development and Non-Technical Skills						
1.1. Non-technical skills (communication, teamwork, resilience, emotional intelligence)	4.51 ± 0.66	0.93	0.15	4.41 ± 0.67	0.88	0.15
1.2. Continuing education in perioperative nursing care	4.62 ± 0.53	0.95	0.11	4.54 ± 0.56	0.97	0.12
1.3. Interdisciplinary briefing and debriefing in perioperative emergencies	---	---	---	4.62 ± 0.52	0.97	0.11
1.4. Core competencies for the expert perioperative nurse	---	---	---	4.28 ± 0.75	0.77	0.18
2. Person-Centered and Culturally Sensitive Perioperative Care						
2.1. Adaptation of perioperative care to diverse cultures	4.33 ± 0.69	0.79	0.16	4.07 ± 0.76	0.74	0.19
2.2. Adaptation of perioperative care for pediatric patients	4.57 ± 0.56	0.93	0.12	4.42 ± 0.58	0.91	0.13
2.3. Adaptation of perioperative care for pregnant women	4.46 ± 0.61	0.89	0.14	4.30 ± 0.58	0.91	0.14
2.4. Adaptation of perioperative care for older adults	4.45 ± 0.59	0.91	0.13	4.41 ± 0.60	0.91	0.14
2.5. Adaptation of perioperative care for individuals with cognitive impairment	4.44 ± 0.60	0.89	0.14	4.38 ± 0.62	0.88	0.14
2.6. Adaptation of perioperative care for individuals with palliative needs	---	---	---	4.35 ± 0.59	0.91	0.14
2.7. Person and family-centered nursing care	4.34 ± 0.82	0.72	0.19	4.30 ± 0.63	0.83	0.15
3. Organizational Aspects and Work Environment						
3.1. Models of perioperative care organization	4.40 ± 0.60	0.89	0.14	4.35 ± 0.70	0.83	0.16
3.2. Leadership Styles	4.34 ± 0.70	0.79	0.16	4.33 ± 0.85	0.77	0.2
3.3. Micromanagement in the operating room	---	---	---	4.26 ± 0.70	0.83	0.16
3.4. Nurse-to-patient ratios in the perioperative setting	4.52 ± 0.71	0.84	0.16	4.36 ± 0.79	0.77	0.18
3.5. Organizational climate	4.46 ± 0.64	0.84	0.14	4.51 ± 0.63	0.91	0.14
3.6. Workplace violence/bullying in the perioperative environment	4.49 ± 0.66	0.82	0.15	4.54 ± 0.66	0.88	0.15
3.7. Interdisciplinary collaboration	4.59 ± 0.56	0.93	0.12	4.51 ± 0.68	0.86	0.15
3.8. Environmental sustainability in the operating room	---	---	---	4.41 ± 0.63	0.91	0.14
3.9. Availability of standard perioperative protocols/procedures	---	---	---	4.51 ± 0.59	0.94	0.13
3.10. Nurse burnout and professional satisfaction	---	---	---	4.74 ± 0.56	0.97	0.12
3.11. Perioperative nursing-sensitive indicators	---	---	---	4.33 ± 0.76	0.80	0.18
3.12. Continuous quality improvement projects in perioperative care	---	---	---	4.51 ± 0.50	0.97	0.11
4. Patient and Team Safety in the Perioperative Setting						
4.1. Patient safety in the perioperative environment	4.71 ± 0.53	0.93	0.11	4.68 ± 0.47	1.00	0.1
4.2. Perioperative team safety (occupational risks, such as noise, musculoskeletal injuries)	4.66 ± 0.50	0.98	0.11	4.65 ± 0.51	0.97	0.11
4.3. Intraoperative materials safety (e.g., patient positioning in the operating room)	4.66 ± 0.52	0.95	0.11	4.62 ± 0.49	1.00	0.11
4.4. Medication safety	4.64 ± 0.57	0.91	0.12	4.48 ± 0.63	0.88	0.14
4.5. Pressure ulcer prevention	4.43 ± 0.69	0.77	0.16	4.30 ± 0.58	0.91	0.14
4.6. Perioperative fall prevention	---	---	---	4.16 ± 0.61	0.83	0.15
4.7. Surgical Safety Checklist	---	---	---	4.32 ± 0.68	0.83	0.16
4.8. Perioperative emergencies/hemodynamic instability	---	---	---	4.58 ± 0.55	0.97	0.12
4.9. Maintenance of perioperative normothermia	4.44 ± 0.68	0.84	0.15	4.29 ± 0.60	0.88	0.14
4.10. Promotion of surgical conscience (advocating for patient safety in the surgical environment)	4.53 ± 0.59	0.91	0.13	4.45 ± 0.61	0.91	0.14
4.11. Use of surgical caps and shoe covers in specific perioperative practices	3.95 ± 0.75	0.49	0.19	---	---	---
4.12. Perioperative thromboembolism prevention	---	---	---	4.32 ± 0.58	0.94	0.13
4.13. Contingency planning/disaster response	---	---	---	4.62 ± 0.57	0.94	0.12
4.14. Preoperative assessment/visit	---	---	---	4.28 ± 0.66	0.83	0.15
4.15. Healthcare-associated infections (HAIs)	---	---	---	4.54 ± 0.56	0.97	0.12
5. Digital Innovation						
5.1. Integrated nursing information systems	4.36 ± 0.61	0.86	0.14	4.28 ± 0.67	0.88	0.16
5.2. Technology to support clinical decision-making	4.32 ± 0.64	0.82	0.15	4.23 ± 0.60	0.88	0.14
5.3. Use of artificial intelligence (AI) and innovation in perioperative nursing	---	---	---	4.13 ± 0.66	0.77	0.16
5.4. Accessibility of perioperative care	4.28 ± 0.73	0.84	0.17	4.14 ± 0.67	0.77	0.16
6. Patient Outcomes and Quality of Perioperative Care						
6.1. Patient/family/caregiver satisfaction with the surgical experience	4.48 ± 0.65	0.89	0.15	4.43 ± 0.60	0.91	0.14
6.2. Recovery of self-care	4.47 ± 0.73	0.89	0.16	4.26 ± 0.70	0.80	0.16
6.3. Recovery of functional capacity	4.51 ± 0.71	0.91	0.16	4.33 ± 0.68	0.86	0.16
6.4. Prevention of adverse events	4.64 ± 0.53	0.95	0.11	4.49 ± 0.60	0.94	0.13
6.5. Symptom control	4.51 ± 0.59	0.95	0.13	4.36 ± 0.59	0.91	0.14
6.6. Acute pain management	---	---	---	4.61 ± 0.52	0.97	0.11
6.7. Influence of the operating room’s sound environment on the patient’s subjective experience	---	---	---	4.42 ± 0.58	0.91	0.13

* Items are listed in thematic domain and numerical code order, not by priority ranking.

## Data Availability

The datasets generated during and/or analyzed during the current study are available from the corresponding author upon reasonable request due to privacy or ethical restrictions.

## Supplementary Materials

The following supporting information can be downloaded at: https://www.mdpi.com/article/10.3390/nursrep16080273/s1, File S1: Guideline for Reporting Delphi Studies.

## Public Involvement Statement

No public involvement in any aspect of this research.

## Guidelines and Standards Statement

This manuscript was drafted against the DELPHISTAR (Guideline for Reporting Delphi Studies).
